# First Report on the Occurrence and Antibiotic Resistance Profile of Colistin-Resistant *Escherichia coli* in Raw Beef and Cow Feces in Vietnam

**DOI:** 10.3390/microorganisms12071305

**Published:** 2024-06-27

**Authors:** Hoang Minh Duc, Tran Thi Khanh Hoa, Nguyen Van Thang, Hoang Minh Son

**Affiliations:** 1 Department of Veterinary Public Health, Faculty of Veterinary Medicine, Vietnam National University of Agriculture Trau Quy, Gia Lam, Hanoi 12400, Vietnam; 2 Veterinary Hospital, Faculty of Veterinary Medicine, Vietnam National University of Agriculture Trau Quy, Gia Lam, Hanoi 12400, Vietnam; 3 Department of Anatomy and Histology, Faculty of Veterinary Medicine, Vietnam National University of Agriculture, Trau Quy, Gia Lam, Hanoi 12400, Vietnam

**Keywords:** *Escherichia coli*, colistin, ESBL, raw beef, cow feces

## Abstract

Colistin-resistant *Escherichia coli* (COE) has been recently recognized as a serious threat to animal and human health. This study aimed to determine the prevalence and antibiotic resistance profile of COE isolated from raw beef and cow feces in Vietnam. Our results showed that 16% (16/100) and 32% (32/100) of raw beef and cow feces samples were positive for COE, respectively. A total of 48 COE strains were isolated, with 16 originating from raw beef and 32 from cow feces samples. The antibiotic susceptibility test revealed that the COE isolates were highly resistant to ampicillin, tetracycline, florfenicol, trimethoprim/sulfamethoxazole, streptomycin, and nalidixic acid, with resistance rates ranging from 66.67% to 87.5%. In addition, 87.5% of the isolates were identified to be multidrug-resistant strains. Further molecular characterization indicated that all COE isolates carried the *mcr-1* gene, with 16 of them also harboring *bla*_CTX-M-55_ genes. Taken together, the findings in this study demonstrate that raw beef and cow feces are important sources of COE, which can be potentially transmitted to humans through the food chain.

## 1. Introduction

*Escherichia coli* (*E. coli*) is a commensal bacterium colonizing the intestinal tract of humans and warm-blooded animals [1]. Therefore, this bacterium can easily contaminate meat during slaughter [2]. As a result, *E. coli* is usually utilized as an indicator of fecal contamination in meat [3]. In addition, *E. coli* is also known as an effective indicator microorganism for antibiotic resistance (AMR) monitoring in animals, humans, and food [4,5] since this bacterium is capable of acquiring resistant genes and transferring them to other commensal and pathogenic bacteria in the intestinal tract [6]. The ease of isolation and cost-effectiveness further justify the selection of *E. coli* for AMR surveillance programs [5,7,8,9].

AMR poses a significant threat to public health, economic development, and food safety worldwide [10,11]. According to the World Health Organization (WHO), AMR is responsible for at least 700,000 annual deaths worldwide [12]. Alarmingly, this number could escalate to a staggering 10 million deaths per year by 2050 [10,12]. Recently, livestock has been increasingly recognized as a reservoir for antibiotic-resistant bacteria that can be transferred to humans through the consumption of contaminated meat products. In Vietnam, antibiotics have been widely used in livestock for disease treatment, prevention, and growth promotion, leading to the development of AMR. It was recently reported that in Vietnam (2016), approximately 77.4 and 286.6 mg of antibiotics were added to feed to produce 1 kg of chickens and pigs, respectively [13]. The most commonly used antibiotics in the chicken industry in Vietnam were bacitracin, chlortetracycline, colistin, and enramycin [13]. In the pig industry, florfenicol, chlortetracycline, colistin, and bacitracin were frequently selected antibiotics [13].

Colistin (polymyxin E) has been classified by the WHO in the group of “Highest Priority Critically Important Antimicrobials for Human Medicine” as this antibiotic is considered to be the last-resort antimicrobial for the treatment of life-threatening infections in humans caused by multidrug-resistant Gram-negative bacteria [11,14]. However, in many countries [15,16,17], including Vietnam [18], colistin has been used extensively for decades for livestock prophylactic, therapeutic, and even growth promotion purposes. Consequently, the prevalence of colistin-resistant *E. coli* (COE) isolates in meat and food-producing animals has recently been reported worldwide [11,19,20,21,22,23].

*β*-lactam antibiotics have been widely used in both human and animal healthcare sectors [24]. WHO has also categorized the third-generation cephalosporins as “Highest Priority Critically Important Antimicrobials for human medicine” [25]. Extended-spectrum *β*-lactamases (ESBLs) are a type of *β*-lactamase that confer bacterial resistance to the first, second, and third-generation cephalosporins and aztreonam [26]. The genes encoding ESBLs are highly diverse and can be grouped into several families, including *bla*_TEM_, *bla*_SHV_, and *bla*_CTX-M_ [27]. The *bla*_CTX-M_ is commonly found in *E. coli* isolates of animal origin [28]. Plasmids and mobile genetic elements have been known to be the important factors contributing to the widespread distribution of *bla*_CTX-M_ [29]. The *bla*_CTX-M_ family is mainly divided into five groups: *bla*_CTX-M-1_, *bla*_CTX-M-2_, *bla*_CTX-M-8_, *bla*_CTX-M-9_ and *bla*_CTX-M-25_ [30]. These subtypes have been recently reported to coexist with other antibiotic-resistant genes, including colistin-resistant genes [31,32].

According to Vietnam’s General Statistical Office (GSO) and Department of Livestock Production (DLP), Vietnam had approximately 6.35 million cattle heads in 2022, with 95% being beef cattle. It was estimated by the Agriculture Organization of the United Nations (FAO) that the average beef consumption of Vietnamese in 2022 was around 8.5 kg per capita per year, which is twice as high as China and six times higher than Thailand [33]. To date, there is no report on the occurrence and antibiotic resistance profile of COE isolates from raw beef and cow feces in Vietnam. The aim of this study was to determine the prevalence and antibiotic resistance profile of COE isolates from raw beef and cow feces collected in Hanoi, Vietnam.

## 2. Materials and Methods

### 2.1. Isolation and Identification of COE

A total of 100 raw beef and 100 cow feces samples were randomly obtained from retail markets and beef cattle farms in Hanoi, Vietnam, from 2022 to 2023 for the isolation of COE. Briefly, 25 g of each sample was homogenized with 225 mL of Buffered Peptone Water (BPW, Oxoid Ltd., Hants, UK) and incubated at 37 °C for 18–24 h. The enriched sample was subsequently streaked onto MacConkey Agar (Oxoid Ltd., Hants, UK) supplemented with 2 mg/L of colistin (Sigma-Aldrich, St. Louis, MO, USA) and incubated at 37 °C for 24 h. Following incubation, presumptive colonies showing typical *E. coli* morphology (pink, flat, and dry) were picked up and re-streaked on Eosin Methylene Blue agar (EMB, Oxoid Ltd., Hants, UK) containing 2 mg/L of colistin and incubated at 37 °C for 24 h. The next day, up to three presumptive colonies of *E. coli* on EMB agar (black centered and flat with metallic green sheen) were grown into Brain Heart Infusion (BHI, Oxoid Ltd., Hants, UK) for biochemical confirmation using API-20E kit (bioMérieux, Marcy l’Etoile, France). Afterward, *E. coli* isolates were preserved at −86 °C for further use.

### 2.2. Antibiotic Susceptibility Test

Verified *E. coli* isolates were tested for their susceptibility to 15 different antibiotics using broth dilution methods according to the guidelines of the Clinical and Laboratory Standards Institute (CLSI) [34]. Antibiotics used for this study include ampicillin, tetracycline, streptomycin, gentamicin, colistin, azithromycin, trimethoprim/sulfamethoxazole, florfenicol, meropenem, cefotaxime, cefoxitin, cefepime, ceftazidime, ciprofloxacin, and nalidixic acid. *E. coli* strain ATCC 25922 served as a quality control strain. The minimum inhibitory concentration (MIC) was determined to be the lowest concentration of an antimicrobial that inhibited the visible growth of the isolate tested. Multidrug-resistant strains were defined as isolates resistant to at least one antibiotic from three or more antibiotic classes.

*E. coli* isolates resistant to cefotaxime and/or ceftazidime were considered as presumptive ESBL producers, and their ESBL-producing ability was phenotypically confirmed using a CLSI confirmatory ESBL test [34].

### 2.3. Detection of mcr Genes and ESBL Encoding Genes

Phenotypically confirmed colistin-resistant isolates (MIC ≥ 4 mg/L) were subjected to multiplex PCR for the detection of *mcr* genes (*mcr-1*, *mcr-2*, *mcr-3*, *mcr-4*, and *mcr-5*) following the previously described method [35]. The DNA extraction of these isolates was performed using a GeneJet Genomic DNA purification kit (Thermoscientific, Vilnius, Lithuania). Primers used for multiplex PCR amplification of *mcr* genes in this study are displayed in Table 1.

Each PCR reaction was carried out in 25 μL mixtures containing 12.5 µL DreamTaq Green PCR Master Mix (Thermo Fisher Scientific, Waltham, MA, USA), 5.5 µL of deionized water, 0.5 µL of each of each primer (10 µM), and 2 µL DNA template. The PCR amplification steps consist of one cycle of denaturation at 94 °C for 5 min; 25 cycles at 94 °C for 30 s, 58 °C for 90 s, and 72 °C for 1 min; and a final extension at 72 °C for 10 min. The amplified PCR product was separated on a 1.5% agarose gel at 75 V in an electrophoresis system (BioRad Laboratories, Hercules, CA, USA) and visualized under ultraviolet light using a BioRad Molecular Imager^®^ GelDocTM XR (BioRad Laboratories, Hercules, CA, USA).

Colitin-resistant *E. coli* isolates capable of producing ESBL were screened for the presence of genes encoding extended-spectrum *β*-lactamases (ESBL genes) by the multiplex PCR following the method previously described by [37]. The primer pairs used for the detection of ESBL genes are listed in Table 2. The multiplex PCR amplification was carried out under the following conditions: denaturation phase at 95 °C/5 min; 25 cycles with denaturation at 95 °C/30 s, annealing at 60 °C/90 s, extension at 72 °C/90 s followed by a final extension at 68 °C for 10 min. The PCR product was then visualized according to the procedure mentioned above.

For further analysis, PCR amplicons of *bla*_CTX-M-1_ group, and *mcr-1* genes were generated and sequenced by the Sanger method using an Applied Biosystems 3500 genetic analyzer (ABI 3500, Applied Biosystems, Foster City, CA, USA). The open reading frames (ORFs) were predicted and annotated using the BLASTP version 2.15.0 [38] and Resfinder-3.1 server [39].

## 3. Results

### 3.1. Prevalence of COE in Raw Beef and Cow Feces

The results of isolation and identification showed that 16 (16%) out of 100 raw beef samples and 32 (32%) out of 100 cow feces samples were positive for COE. To avoid duplication, only one COE strain from each positive sample was isolated and preserved at −86 °C. A total of 48 isolates, including 16 from raw beef and 32 from cow feces, were subjected to the antimicrobial susceptibility test.

### 3.2. Antimicrobial Susceptibility Profile of COE Isolates

The antimicrobial susceptibility of COE isolates is presented in Table 3. COE isolates from raw beef were highly (93.75–100%) resistant to trimethoprim/sulfamethoxazole, tetracycline, florfenicol, and nalidixic acid, followed by ampicillin, streptomycin, gentamicin, and ciprofloxacin with resistance rates ranging from 62.5–87.5%. Lower resistance rates were observed with cefotaxime, ceftazidime, cefepime, and azithromycin, which had the same rate of 37.5%. None of the COE isolates from raw beef were resistant to cefoxitin and meropenem.

Overall, resistance rates of COE isolates from cow feces to antibiotic tested were lower than those from raw beef. However, the resistance trends of COE isolates from raw beef and cow feces were relatively similar. The highest resistance levels of COE isolates from cow feces were recorded with ampicillin (87.5%), tetracycline (75%), streptomycin (65.63%), florfenicol (62.5%), and trimethoprim/sulfamethoxazole (59.38%). On the contrary, only 3.13% (1/32) of the isolates were resistant to cefoxitin and azithromycin, and no resistance to meropenem was detected.

The results of the ESBL test revealed that 16 (33.33%) of 48 COE isolates were ESBL producers, corresponding to 8% (16/200) of the samples tested. Among these 16 isolates, 10 (31.25%; 10/32) strains were recovered from cow feces samples and 6 (37.5%; 6/16) from raw beef samples. All COE isolates capable of producing ESBL were subjected to multiplex PCR for the detection of ESBL genes.

The findings in Table 4 revealed that all COE isolates from raw beef samples were resistant to at least five antibiotics. Moreover, 68.75% (11/16) and 31.25% (5/16) of them showed resistance to 5–10 and 11–15 antibiotics tested, respectively. Regarding COE isolates from cow feces, resistant rates to 1, 1–5, 6–10, and 11–15 antibiotics tested were 100%, 28.13% (9/32), 59.38% (19/32), and 12.5% (4/32), respectively. MDR rates of COE isolates from raw beef and cow feces samples were 100% and 81.25%, respectively.

Antibiotic resistance patterns of COE isolates are shown in Table 4. COE isolates from raw beef and cow feces samples exhibited 11 and 17 antibiotic resistance patterns, respectively. The predominant resistance pattern for COE isolates from raw beef was AMP-CTX-CAZ-FEP-GEN-STR-AZM-TET-FLO-CST-SXT-NAL-CIP (3 isolates; 18.75%). Meanwhile, AMP-GEN-STR-TET-FLO-CST-SXT-NAL-CIP was most common in COE isolates from cow feces (6 isolates; 18.75%) (Table 4).

### 3.3. Detection of mcr Genes and ESBL Genes

The result of multiplex PCR showed that 48 (100%) COE isolates in this study harbored the *mcr-1* gene. Among them, 16 isolates were also found to carry the *bla*_CTX-M-1_ gene in addition to the *mcr-1* gene. Further molecular characterization by sequencing indicated that the genotype of *bla*_CTX-M-1_ was *bla*_CTX-M-55_ (Table 5). Six COE isolates co-harboring *mcr-1* and *bla*_CTX-M-55_ from raw beef were resistant to 10–13 antibiotics tested. While 10 *mcr-1* and *bla*_CTX-M-55_ positive COE isolates from cow feces exhibited resistance to 6–14 antibiotics tested (Table 5).

## 4. Discussion

The results of our study showed that the prevalence of COE in raw beef and cow feces in Vietnam was 16% and 32%, respectively. These results correlate with a previous study using the same methodological approach that reported that 10% of imported beef was positive for COE [40]. The prevalence of COE in raw beef in this study is higher than in a previous study conducted by Johura et al. (2020) in Egypt [21]. Out of 150 raw beef samples collected in Mansoura city, Egypt, five samples were positive for COE, representing 3.33%. In a study performed in Italia, a total of 17 *E. coli* strains were isolated from 133 beef samples, but none of them were identified as COE [23]. It is worth mentioning that in our study, selective agar (MacConkey supplemented with 2 mg/mL colistin) was used for the isolation of COE, while in the studies from Egypt and Italia, COE isolates were identified from randomly selected *E. coli* isolates from non-selective agar. One of the main advantages of using selective agar for the isolation of COE is the high sensitivity, which means that COE can be recovered even in samples with a low proportion of COE in the total *E. coli* flora, thereby avoiding an underestimation of COE prevalence. This could be one of the reasons for the higher prevalence of COE in raw beef found in this study compared to previous studies. In Vietnam, the prevalence of COE has been recently reported in pork (40%), chicken meat (66%), pig (45.7%) and humans (23.3%) [41,42]. To the best of our knowledge, this is the first report on the prevalence of COE in raw beef and cow feces in Vietnam. Another factor contributing to the variation in COE prevalence is the different geographical locations. 

Previous studies have shown that the prevalence of COE in food and food-producing animals was higher in Asia, Africa and especially Latin America than in Europe and North America [11]. This may be attributed to the widespread use of colistin in animal husbandry for disease treatment, prevention, and growth promotion in developing countries in Asia, Africa and Latin America [18,21,43]. For instance, in Vietnam, colistin is reportedly the most widely used antibiotic in the chicken and pig industries [18]. In contrast, the EU has banned the use of colistin as an animal growth promoter [44], while in the United States, colistin is not permitted for use in food-producing animals [45]. Overall, the relatively high prevalence of COE observed in raw beef and cow feces in this study underscores the importance of implementing a monitoring program for antibiotic usage and antibiotic susceptibility of bacteria in food and livestock in Vietnam.

As colistin is considered to be the last-resort antimicrobial for the treatment of multidrug-resistant Gram-negative bacteria, including carbapenem-resistant pathogens, the potential transmission of COE from animals to humans through the food chain has become one of the major concerns for human health [11,46]. Furthermore, if COE isolates harbor mobile colistin-resistant genes in transmissible plasmids, they can horizontally transfer these resistance genes to other bacteria, especially pathogenic strains, leading to an even more worrisome scenario [47]. In this study, all COE isolates from raw beef and cow feces were found to carry the *mcr-1* gene. Similar results were also recently observed in previous studies showing that *mcr-1* is frequently detected in COE isolates from food and food-producing animals [21,22,48]. The widespread distribution of *mcr-1* is attributed to the fact that *mcr-1* is located on transferable plasmids that can move horizontally between different bacterial species. In addition, previous studies have reported that *mcr-1* can be mobilized with other mobile genetic elements, such as transposons and integrons [48]

The co-existence of *mcr*-1 and ESBL genes, especially in Gram-negative bacteria such as *E. coli*, is a serious public health concern as it may lead to the limitation of therapeutic options [21,49]. In this study, 16 (33.33%; 16/48) COE isolates co-carrying *mcr-1* and *bla*_CTX-M-55_ gene, including 6 isolates originating from raw beef samples and 10 isolates from cow feces samples, were isolated for the first time in Vietnam, and all (100%, 16/16) of them were determined to be multidrug-resistant strains. Among them, nine isolates exhibited resistance to 12 out of 15 antibiotics tested. In particular, one isolate from cow feces was only susceptible to meropenem. These findings in this study are consistent with previous studies reporting a high prevalence of MDR among COE isolates [40,42,50] and the co-existence of *mcr-1* and *bla*_CTX-M_ gene in COE isolates. When characterizing 54 COE isolates co-harboring *mcr-1* and ESBL genes of chicken origin, Shafiq et al. (2021) found that the combination of *mcr-1* and *bla*_CTX-M-55_ was the most common genotype pattern [48]. In a study conducted in Egypt, 0.95% (2/210) of COE isolates from raw beef and read-to-eat beef products simultaneously carried *mcr-1* and *bla*_CTX-M-28_ [21]. The concurrent carriage of *mcr-1* and *bla*_CTX-M_ genes has also been described by Joshi et al., who reported that 3 out of 27 COE isolates from chicken in Napal simultaneously harbored both genes [51]. Similarly, a local survey in South America showed that 14.6% (6/41) of chicken meat samples were contaminated with COE harboring *mcr-1* along with *bla*_CTX-M-2_ or *bla*_CTX-M-8_ [52]. Also, a recent study on the epidemiology of *mcr* genes in *Enterobacteriaceae* isolated from pigs and humans on farms in Thailand found that *bla*_CTX-M-14_ and *bla*_CTX-M-55_ were the predominant genes co-existing with *mcr* genes in COE isolates [50]. Until now, the mechanism behind the co-occurrence of *mcr-1* and ESBL genes in *E. coli* isolates has not been fully understood. The discovery of an IncHI2-type plasmid capable of concurrently carrying the *mcr-1* gene and *bla*_CTX-M-1_ has partly elucidated the genetic mechanism [53]. The emergence of COE harboring both *mcr-1* and *bla*_CTX-M-55_ observed in this study may be due to the overuse and misuse of colistin and cephalosporins alone or in combination in livestock in Vietnam as previous reports have highlighted colistin and cephalosporins (cefotaxime and ceftiofur) were the most commonly used antibiotics for animal husbandry in Vietnam [13,18,54]. In addition, a study conducted by Cuong et al. (2016) in Vietnam revealed that 21.5% and 5.4% of pig and chicken feed contained at least two antibiotics, respectively [13].

## 5. Conclusions

The findings in our study showed a relatively high prevalence of COE in raw beef and cow feces in Hanoi, Vietnam. Out of 48 COE isolates, 87.5% were identified as MDR strains. All COE isolates were found to carry the *mcr-1* gene, with 33.33% also harboring the *bla*_CTX-M-55_ gene. The presence of these isolates in the food chain (raw beef and cow feces) in Vietnam may pose a significant threat to animal and human health, indicating the necessity for future investigation and intervention measures.

## Figures and Tables

**Table 1:** Primer sequences for the detection of *mcr* genes.

Target Gene	Primer	Primer Sequence	Amplicon Size (bp)	Reference
*mcr-1*	*mcr1* f	AGTCCGTTTGTTCTTGTGGC	320	[35]
	*mcr1* r	AGATCCTTGGTCTCGGCTTG		
*mcr-2*	*mcr2* f	CAAGTGTGTTGGTCGCAGTT	715	[35]
	*mcr2* r	TCTAGCCCGACAAGCATACC		
*mcr-3*	*mcr3* f	AAATAAAAATTGTTCCGCTTATG	929	[35]
	*mcr3* r	AATGGAGATCCCCGTTTTT		
*mcr-4*	*mcr4* f	TCACTTTCATCACTGCGTTG	1116	[35]
	*mcr4* r	TTGGTCCATGACTACCAATG		
*mcr-5*	*mcr5* f	ATGCGGTTGTCTGCATTTATC	1644	[36]
	*mcr5* r	TCATTGTGGTTGTCCTTTTCTG		

**Table 2:** Primer sequences for the detection of ESBL genes.

Target Gene	Primer	Primer Sequence	Amplicon Size (bp)
*bla*_TEM_	TEM-F	GGTCGCCGCATACACTATTCTC	372
	TEM-R	TTTTATCCGCCTCCATCCAGTC	
*bla*_SHV_	SHV-F	CCAGCAGGATCTGGTGGACTAC	231
	SHV-R	CCGGGAAGCGCCCTCCAT	
*bla*_CTX-M-1_	CTX-M1-F	GAATTAGAGCGGGAGTCGGG	588
	CTX-M1-R	CACAACCCAGGAAGCAGGC	
*bla*_CTX-M-2_	CTX-M2-F	GATGGCGACGCTACCCC	107
	CTX-M2-R	CAAGCCGACCTCCCGAAC	
*bla*_CTX-M-9_	CTX-M9-F	GTGCAACGGATGATGTTCGC	475
	CTX-M9-R	GAAACGTCTCATCGCCGATC	
*bla*_CTX-M-8/25_	CTX-M8/25-F	GCGACCCGCGCGATAC	186
	CTX-M8/25-R	TGCCGGTTTTATCCCCG	

**Table 3:** Antimicrobial resistance profile of COE isolates.

Antibiotic Class	Antibiotics	Cow Feces (*n* = 32)		Raw Beef (*n* = 16)		Total (*n* = 48)	
		No. Isolates	%	No. Isolates	%	No. Isolates	%
Penicillin	ampicillin	28	87.5	14	87.50	42	87.5
Cephalosporins	cefotaxime	10	31.25	6	37.50	16	33.33
	cefoxitin	1	3.13	0	0.00	1	2.08
	cefepime	10	31.25	6	37.5	16	33.33
	ceftazidime	10	31.25	6	37.5	16	33.33
Cabarpenems	meropenem	0	0	0	0.00	0	0
Tetracyclines	tetracycline	24	75	15	93.75	39	81.25
Phenicols	florfenicol	20	62.5	15	93.75	35	72.92
Polymyxins	colistin	32	100	16	100.00	48	100
Sulfonamides	trimethoprim/sulfamethoxazole	19	59.38	16	100.00	35	72.92
Quinolones	nalidixic acid	17	53.13	15	93.75	32	66.67
Fluoroquinolones	ciprofloxacin	15	46.88	10	62.5	25	52.08
Aminoglycosides	gentamicin	17	53.13	11	68.75	28	58.33
	streptomycin	21	65.63	12	75.00	33	68.75
Macrolides	azithromycin	1	3.13	6	37.50	7	14.58

**Table 4:** Antibiotic resistance patterns of COE isolates.

No. of Antibiotics	Resistance Pattern	No. of *E. coli* Isolates (%)		
		Cow Feces	Raw Beef	Total
1	CST	3 (9.38)	0 (0)	3 (6.25)
2	AMP-CST	2 (6.25)	0 (0)	2 (4.17)
	TET-CST	1 (3.13)	0 (0)	1 (2.08)
3	AMP-CST-NAL	1 (3.13)	0 (0)	1 (2.08)
4	AMP-CST-NAL-CIP	2 (6.25)	0 (0)	2 (4.17)
5	AMP-TET-FLO-CST-SXT	0 (0)	1 (6.25)	1 (2.08)
6	AMP-CTX-CAZ-FEP-TET-CST	2 (6.25)	0 (0)	2 (4.17)
	AMP-STR-TET-FLO-CST-SXT	2 (6.25)	0 (0)	2 (4.17)
	AMP-TET-FLO-CST-SXT-NAL	0 (0)	2 (12.5)	2 (4.17)
	AMP-STR-TET-CST-SXT-NAL	0 (0)	1 (6.25)	1 (2.08)
7	AMP-STR-TET-FLO-CST-SXT-CIP	1 (3.13)	0 (0)	1 (2.08)
	AMP-GEN-STR-TET-FLO-CST-SXT	1 (3.13)	0 (0)	1 (2.08)
8	AMP-GEN-STR-TET-FLO-CST-SXT-NAL	2 (6.25)	1 (6.25)	3 (6.25)
	AMP-STR-TET-FLO-CST-SXT-NAL-CIP	1 (3.13)	0 (0)	1 (2.08)
	GEN-STR-TET-FLO-CST-SXT-NAL-CIP	0 (0)	2 (12.5)	2 (4.17)
9	AMP-GEN-STR-TET-FLO-CST-SXT-NAL-CIP	6 (18.75)	2 (12.5)	8 (16.67)
	AMP-CTX-CAZ-FEP-GEN-STR-TET-FLO-CST	1 (3.13)	0 (0)	1 (2.08)
10	AMP-CTX-CAZ-FEP-GEN-STR-TET-FLO-CST-SXT	2 (6.25)	0 (0)	2 (4.17)
	AMP-CTX-CAZ-FEP-GEN-STR-TET-CST-NAL-CIP	1 (3.13)	0 (0)	1 (2.08)
	AMP-GEN-STR-AZM-TET-FLO-CST-SXT-NAL-CIP	0 (0)	1 (6.25)	1 (2.08)
	AMP-CTX-CAZ-FEP-GEN-FLO-CST-SXT-NAL-CIP	0 (0)	1 (6.25)	1 (2.08)
12	AMP-CTX-CAZ-FEP-GEN-STR-TET-FLO-CST-SXT-NAL-CIP	3 (9.38)	0 (0)	3 (6.25)
	AMP-CTX-CAZ-FEP-STR-AZM-TET-FLO-CST-SXT-NAL-CIP	0 (0)	1 (6.25)	1 (2.08)
	AMP-CTX-CAZ-FEP-GEN-STR-AZM-TET-FLO-CST-SXT-NAL	0 (0)	1 (6.25)	1 (2.08)
13	AMP-CTX-CAZ-FEP-GEN-STR-AZM-TET-FLO-CST-SXT-NAL-CIP	0 (0)	3 (18.75)	3 (6.25)
14	AMP-CTX-FOX-CAZ-FEP-GEN-STR-AZM-TET-FLO-CST-SXT-NAL-CIP	1 (3.13)	0 (0)	1 (2.08)

AMP—ampicillin; CTX—cefotaxime; FOX—cefoxitin; FEP—cefepime; CAZ—ceftazidime; TET—tetracycline; STR—streptomycin; GEN—gentamicin; AZM—azithromycin; FLO—florfenicol; SXT—Trimethoprim/sulfamethoxazole; CIP—ciprofloxacin; NAL—nalidixic acid; CST—colistin.

**Table 5:** Phenotypic and genotypic antibiotic resistance of COE isolates capable of producing ESBL.

Source	Strain ID	ESBL Gene	*mcr* Gene	Resistance Phenotype
Cow feces	EPBC3	*bla*_CTX-M-55_	*mcr-1*	AMP-CTX-CAZ-FEP-GEN-STR-TET-FLO-CST-SXT
	EPBC5	*bla*_CTX-M-55_	*mcr-1*	AMP-CTX-CAZ-FEP-TET-CST
	EPBC7	*bla*_CTX-M-55_	*mcr-1*	AMP-CTX-CAZ-FEP-GEN-STR-TET-FLO-CST-SXT-NAL-CIP
	EPBC10	*bla*_CTX-M-55_	*mcr-1*	AMP-CTX-CAZ-FEP-GEN-STR-TET-FLO-CST-SXT-NAL-CIP
	EPBC19	*bla*_CTX-M-55_	*mcr-1*	AMP-CTX-CAZ-FEP-GEN-STR-TET-FLO-CST-SXT
	EPBC20	*bla*_CTX-M-55_	*mcr-1*	AMP-CTX-CAZ-FEP-GEN-STR-TET-FLO-CST
	EPBC22	*bla*_CTX-M-55_	*mcr-1*	AMP-CTX-CAZ-FEP-GEN-STR-TET-FLO-CST-SXT-NAL-CIP
	EPBC24	*bla*_CTX-M-55_	*mcr-1*	AMP-CTX-CAZ-FEP-TET-CST
	EPBC26	*bla*_CTX-M-55_	*mcr-1*	AMP-CTX-CAZ-FEP-GEN-STR-TET-CST-NAL-CIP
	EPBC31	*bla*_CTX-M-55_	*mcr-1*	AMP-CTX-FOX-CAZ-FEP-GEN-STR-AZM-TET-FLO-CST-SXT-NAL-CIP
Raw beef	ETBC12	*bla*_CTX-M-55_	*mcr-1*	AMP-CTX-CAZ-FEP-STR-AZM-TET-FLO-CST-SXT-NAL-CIP
	ETBC15	*bla*_CTX-M-55_	*mcr-1*	AMP-CTX-CAZ-FEP-GEN-STR-AZM-TET-FLO-CST-SXT-NAL-CIP
	ETBC18	*bla*_CTX-M-55_	*mcr-1*	AMP-CTX-CAZ-FEP-GEN-STR-AZM-TET-FLO-CST-SXT-NAL-CIP
	ETBC19	*bla*_CTX-M-55_	*mcr-1*	AMP-CTX-CAZ-FEP-GEN-STR-AZM-TET-FLO-CST-SXT-NAL-CIP
	ETBC22	*bla*_CTX-M-55_	*mcr-1*	AMP-CTX-CAZ-FEP-GEN-STR-AZM-TET-FLO-CST-SXT-NAL
	ETBC23	*bla*_CTX-M-55_	*mcr-1*	AMP-CTX-CAZ-FEP-GEN-FLO-CST-SXT-NAL-CIP

## Data Availability

The original contributions presented in the study are included in the article, further inquiries can be directed to the corresponding author.
